# Reducing inequalities in cardiovascular disease: focus on marginalized populations considering ethnicity and race

**DOI:** 10.1016/j.lanepe.2025.101371

**Published:** 2025-08-21

**Authors:** Sonia S. Anand, Sujane Kandasamy, Miles Marchand, Maryam Kavousi, Martha Gulati, John Deanfield, Arshed A. Quyyumi

**Affiliations:** a Chanchlani Research Centre, Department of Medicine, McMaster University, Hamilton, Ontario, Canada; b Mary Heersink School of Global Health and Social Medicine, McMaster University, Hamilton, Ontario, Canada; c Population Health Research Institute, Hamilton Health Sciences, McMaster University, Hamilton, Ontario, Canada; d Department of Medicine, McMaster University, Hamilton, Ontario, Canada; e Department of Child and Youth Studies, Brock University, St Catharines, Ontario, Canada; f Division of Cardiology, University of British Columbia, Vancouver, British Columbia, Canada; g Member of the Syilx Okanagan First Nation, British Columbia, Canada; h Department of Epidemiology, Erasmus MC, University Medical Center Rotterdam, Rotterdam, the Netherlands; i Barbara Streisand Women's Heart Center, Cedars-Sinai Smidt Heart Institute, Los Angeles, CA, USA; j The Baim Institute for Clinical Research, Boston, MA, USA; k Institute of Cardiovascular Sciences, University College London, UK; l Emory Clinical Cardiovascular Research Institute, Division of Cardiology, Department of Medicine, Emory University School of Medicine, Atlanta, GA, USA; m Department of Epidemiology, Rollins School of Public Health, Emory University, Atlanta, GA, USA

**Keywords:** Race, Ethnicity, Cardiovascular disease, Health equity

## Abstract

Cardiovascular disease (CVD) and its risk factors are more prevalent among traditionally marginalized racial, ethnic, and Indigenous groups. These populations also often face greater barriers to accessing cardiovascular health care, further contributing to the health equity gap. To address the challenge of inequalities and disparities in cardiovascular health outcomes, the Lancet Regional Health—Europe convened experts to evaluate the current state of knowledge on inequalities and disparities in cardiovascular health among marginalized populations and propose recommendations to address these disparities. This Series paper aims to review disparities in CVD referring to coronary heart disease and stroke, based on race, ethnicity, ancestry, and Indigeneity emphasizing the intersection of these factors with sex, gender, and socioeconomic status (SES) across Europe and North America. These regions were chosen as they have well established health-care systems, with persistent, and in some regions widening, disparities in cardiovascular health and outcomes. Ethnicity and race should be measured in a standardized manner in health-care administrative databases to identify high risk groups who might need focused programmes to improve health-care access and to address bias and inequities in care. Strategies that policymakers, health-care professionals, and advocacy groups can use to advance cardiovascular health equity include improving access to health-care systems and research for high-risk communities, fostering trust between these communities and public health providers, and enhancing the delivery of evidence-based therapies for the prevention and treatment of CVD.

## Introduction

**Fig. 1:**
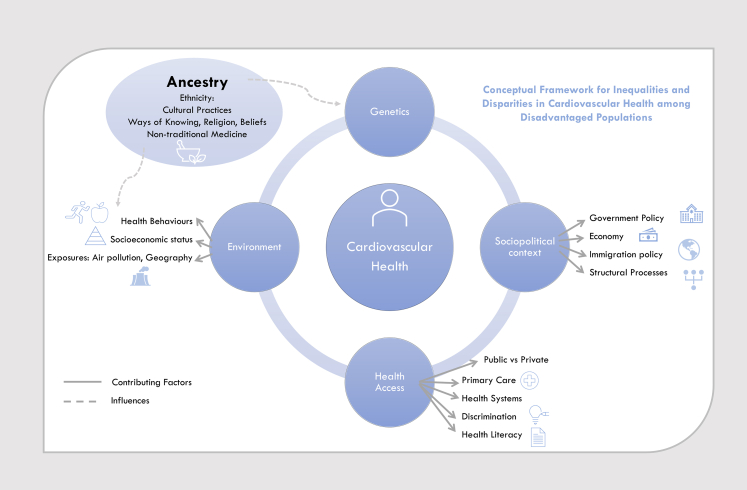
**Framework for understanding inequalities and disparities in cardiovascular health for marginalized populations.** Conceptual framework illustrates multifaceted and intersecting determinants contributing to inequalities in cardiovascular health among disadvantaged populations. Cardiovascular health is positioned at the center, as the primary outcome of interest, while genetics, sociopolitical context, health access, environment, and ancestry are depicted as key influencing domains. Circular connections between domains signify mutual influence rather than unidirectional relationship. Dashed arrows represent indirect influences, while solid arrows indicate contributing factors extending from the conceptual model. This emphasizes that various structural and environmental determinants shape cardiovascular health and not a directional effect away from health.

Cardiovascular disease (CVD) and its risk factors are more prevalent among traditionally marginalized ethnic, racial, and Indigenous groups. These populations also often face greater barriers to accessing cardiovascular health care, further contributing to the health equity gap. Ethnicity, race, and ancestry are often used interchangeably in health research causing confusion^1^; the definitions we use are found in Panel 1. Equity initiatives in cardiovascular health seek to characterize disparities and to develop targeted initiatives to improve the health status of high-risk groups, especially those who have been historically underserved or marginalized. Intersecting factors can compound health disparities, leading to more pronounced inequities for certain groups. The combination of ethnic, race or ancestral background, with gender, and social disadvantage can create unique vulnerabilities that exacerbate cardiovascular risks and underline the importance of considering these intersecting social determinants in public health, research, and policy. A comprehensive approach that includes the social context, including socioeconomic status (SES), gender, cultural practices, historical context, and structural inequities, provides a more accurate and holistic understanding of the root causes of health disparities and how they can be addressed^4^ (Fig. 1).

**Search strategy and selection criteria**

We conducted a structured MEDLINE search via OVID from database inception to July 21, 2024. A top-up search was conducted on April 4, 2025 to capture any updates. Search terms, which are listed in Appendix S1, were developed in collaboration with a McMaster University information specialist to capture titles that reflect ethnic and racial CV disparities—including screening, diagnosis, and management—within specific geographic regions. Titles and abstracts were screened by experts in the field and within selected geographic regions, and full-text papers were included if the focus was on CV disparities based on ethnicity/race and within assigned geographic regions. We also supplemented this search with a hand search of reference lists of included studies, review of subsequent publications related to parent studies, and expert review. To identify publications on Indigenous CV health disparities, a similar search strategy was applied, with additional search term filters for Indigenous peoples in Canada, USA, Mexico, Central America and Europe. The search was not limited by year but focused on papers published in the last 10-years.

**Key messages**

**Trends**

• Within countries and regions, the distribution of cardiovascular disease (CVD) and traditional cardiovascular (CV) risk factors vary by ethnicity and race in terms of prevalence, severity, and outcomes, with some groups experiencing disproportionately higher burdens of CVD and associated risk factors.
• The patterns of CVD and CV risk factors are also influenced by socioeconomic factors which are key intersecting factors that can mitigate some of the impact of ethnicity and race when socioeconomic status (SES) is high and compound the impact of ethnicity and race when it is low.
• Gender is a key intersecting factor, as women from minority ethnic groups of low SES have the greatest burden of CV risk factors such as hypertension, diabetes, and obesity in both high- and low-income settings and poorest health outcomes, due to barriers limiting healthcare access, systemic discrimination, and socioeconomic constraints.
• CVD variation by ethnicity and race are also influenced by access to healthcare, where in immigrants to countries within Europe and North America, especially those with low SES, language barriers or those who face discrimination, have lower healthcare access, due to factors including lack of culturally competent care, systemic exclusion, and restrictive insurance structures. Non-immigrant, ethnic minority Americans in the US without health insurance also face similar barriers to access.
• Some countries such as Costa Rica's universal offering of primary care serves to more than 90% of the country and which is community-oriented integrated prevention focussed, reduces the impact of poverty and minimizes the CV health equity gap.
• Indigenous peoples, irrespective of region, continue to be affected by acculturation, marginalization and other impacts of colonialism that have disrupted their traditional, healthy lifestyles. Populations of Indigenous peoples who have maintained or restored their traditional lifestyles by emphasizing traditional diets, active living, and community-based health initiatives has displayed improved CV health irrespective of the barriers to care they face.

**Actions**

• Governments should monitor trends of CV health by ethnicity, race, and ancestry using direct measures such as self-reported data if it can be collected in a safe, unstigmatizing manner.
• Where high risk communities are identified, targeted screening and treatment programs should be undertaken, e.g., screening and control of blood pressure among Black men,
• Where effective CV medications are expensive and not covered by insurance, public health programs should screen and provide low-cost treatment with WHO Best Buys, together with health systems strengthening programs.
• Clinicians should be aware of the CVD and CV risk factor distribution by ethnicity, race, and ancestry e.g., diabetes rates among South Asian and Indigenous populations and routinely screen for these conditions
• Community-based programs could focus on healthy active living promoting culturally tailored healthy foods and activity programs across the life-course.

Panel 1 **Note on ethnicity, race, and ancestry and social disadvantage.**

Ethnicity, race, and ancestry are classification variables often misused in research due to a lack of consensus definition across geographic regions.^1^ Inconsistent terminology across health literature leads to varying interpretations by researchers, participants, and society.

***Ethnicity***, refers to groupings based on shared cultural practices such as language, religion, dietary habits, and nationality.^1^ Ethnicity may also reflect common ancestry or geographic origin, and in some instances ethnic grouping can reflect a unique biological response to a treatment or higher prevalence of a disease.^1^ Ethnicity data has been systematically collected in the United Kingdom (UK) Census since 1991, but standardized collection is rare or absent in Canada, Europe, and Central America or Mexico.

***Race*** is defined as a social construct that categorizes people based on physical differences, like skin color. Race is a useful proxy for social inequities driven by racism. While race is widely used in the US, including in census data, it is less common in Canada, Mexico, or Europe.

***Ancestry*** can be used to classify individuals based on shared geographic, genealogic, or genetic biological decent.^1^

***Indigeneity:*** Indigeneity is distinct from race, ethnicity, and ancestry and refers to the cultural, social, and historical ties that first peoples have to their ancestral lands, as well as their distinct cultural practices, languages, and knowledge systems.

***Social disadvantage*** refers to the unequal distribution of adverse socioeconomic conditions by age, sex, and ethnicity.^2^ This can result in ***marginalization***, where individuals suffering from these inequalities feel excluded due to their identity, associations, experiences, and environment.

Summary: These classifications not only influence disease risk but shape individuals' health, perception of healthcare, and their experiences of social disadvantage and marginalization.^3^

In this Series paper, we aim to examine epidemiologic trends in CVD and cardiovascular risk factors across ethnic, race, and Indigenous groups in Europe and North America, considering the intersection of gender, social disadvantage, health-care access, and resulting inequalities. We focus on these regions which have well established health-care systems but also persisting disparities in cardiovascular health and outcomes. The United Kingdom (UK) is considered separately from continental Europe in this analysis to reflect its unique historical and healthcare context. Specifically, the UK has had a longer and more pronounced migration history from the Indian subcontinent and the Caribbean than most other European countries, shaping its demographic composition, research infrastructure, and policy responses in ways that differ from other nations on the continent such as France and Spain. For parsimony, we use “continental Europe” to refer to European countries excluding the UK and Ireland and acknowledge substantial heterogeneity in health-care systems between European countries exists.

Within North America, Canada and the United States are considered separately due to two key reasons, their fundamentally different health-care systems, and the distinct ways in which race and ethnicity are classified and operationalized in health research and policy. Mexico, Central America, and the Caribbean are grouped together because they are geographically close and have been shaped by similarities in their colonial histories. We also give particular consideration to the challenges faced by Indigenous populations^5^ in Europe and North America and suggest ways forward in reducing disparities in cardiovascular health (Table 1).

**Table 1:** Major similarities and differences among regions.

	Europe	UK	USA	Canada	Mexico, Central America, and the Caribbean
Standardized ascertainment of ethnicity, race, or Indigeneity	Inconsistent ethnicity or race collection^6^	Yes, ethnicity on Census^7^ Yes, ethnicity on healthcare records^8^	Yes, race on Census^9^ Race ± ethnicity on healthcare records by variation by program and state	Ethnicity, visible minority status, and Indigenous on Census^10^ Not collected on healthcare records	Not race but some ethnicities e.g., Indigenous CARICOM is initiating self-reported ethnicity for Caribbean^11^
Ethnic and Indigenous makeup	Majority White Ethnic minority groups from South Asian, African, and Middle Eastern descent are growing using proxy measures of ethnicity i.e., country of origin and health surveys^6^ Largest non-EU citizen groups are Ukrainian, Turkish, and Moroccan^12^ Indigenous as First People in Northern European countries Northern Scandinavia, Greenlandic Inuit, and Russia^13^, ^14^, ^15^	Majority White 23% non-White with largest non-White groups include South Asians, Black, and Mixed populations^8^	Majority White 40% are non-White; largest non-White groups include Hispanic or Latino, Black or African Americans, and Asians^16^ American Indian/Hawaiian^17^^,^^18^	Majority White 26·5% of population are non-White South Asian, Chinese, Black, Filipino, and Arab populations are the largest non-White ethnic groups^10^ Indigenous people make up 5% of Canada^19^	Mixed populations Mexico: Varying proportions of Indigenous, European, and African ancestry; diversity across countries Mestizo is 62%, predominantly Indigenous 21%, Indigenous 7%, other 10% (mostly European)^20^ Central American Caribbean: Diverse mixed population of African, South Asian, Indigenous, and European^11^
Groups with highest CVD mortality	Eastern e.g., Russia, Ukraine, and Bulgaria) and Central European countries (higher than in Northern, Southern, and Western European countries,^21^ no good ethnic specific data	Black African, Caribbean, South Asian^7^	American Indian and Alaskan Natives and Non-Hispanic Black individuals^16^^,^^22^	Indigenous populations and South Asians^2^	Caribbean population, South Asians experience high CHD, and African origin have high stroke burden^23^^,^^24^ Scarce data from Indigenous only populations^25^
Groups with lowest CVD mortality	Western European countries (e.g., France and Switzerland) Within HIC Western countries,^21^ ethnic minorities have higher CVD^3^^,^^26^^,^^27^	White British populations^7^	Chinese Americans^9^	Chinese and White populations^2^	Data varies across region
Common risk factors	Obesity^28^ Hypertension^29^ Diabetes Low physical activity^21^ Smoking^21^	Obesity^30^ Hypertension^7^ High cholesterol^31^ Diabetes^7^ Low physical activity^31^ Smoking^30^ ∗Significant ethnic variation reported	Obesity^32^ Hypertension^32^ High cholesterol^32^ Diabetes^32^ Low physical activity^32^ Smoking^32^ ∗Higher burden among black, Hispanic and American Indians	Obesity^33^ Hypertension^33^ High cholesterol^34^^,^^35^ Diabetes^34^^,^^35^ Low physical activity^36^ Smoking^36^ ∗Significant ethnic variation reported	Obesity^37^ Hypertension^38^ Diabetes^37^
Healthcare system type	Varies by country; mix of universal healthcare coverage funded by taxation or social insurance and private insurance^39^ Results in variations in CV care delivery^39^	Universal healthcare system (NHS) funded by taxation. South Asians and Black population receive fewer interventions for heart disease^40^^,^^41^	Mixed system with private and public providers; lacks universal coverage	Universal healthcare system funded by taxation; publicly funded and administered^42^	Varies by country; most have mixed public-private systems with varying degrees of access and coverage Costa Rica has universal access and decline in CVD due to health systems reform^43^
Evidence supports	Low SES and gender intersect with ethnicity^3^	Health inequalities apparent (including unequal access to care) between the most (e.g., high burden of risk factors and greater structural barriers) and least deprived communities^30^	Social gradient evidence, lower socioeconomic status higher burden of CV risk factors^44^; Structural racism strong determinant of CVD risk among Black and Native Americans^22^^,^^45^	Social disadvantage and gender intersect with ethnicity and influence CVD rates and risk factors^2^ Unique barriers for reserve based Indigenous peoples^46^	Significant health disparities due to socioeconomic inequality^47^

CARICOM: Caribbean Community; EU: European Union; CHD: Coronary Heart Disease; CVD: Cardiovascular Disease; NHS: National Health Service; CV: Cardiovascular.

### Europe excluding the UK

#### Epidemiology of CVD

The countries of continental Europe are diverse in geography, culture, health policy, and income inequality. To facilitate analysis, we reference the European Society of Cardiology's (ESC) classification of European countries into four cardiovascular risk categories—low, moderate, high, and very high—based on age-standardized CVD mortality rates.^48^ While CVD remains the leading cause of mortality and a major cause of morbidity in Europe, mortality from coronary heart disease (CHD) stroke are higher in central and eastern Europe than in northern, southern and western Europe.^21^ These regional differences are corroborated by the Global Burden of Disease (GBD) 2021 estimates, which show substantial east–west gradients in CVD burden across the continent, including disparities in both mortality and disability-adjusted life years (DALYs).^28^ The proportion of citizens who originate from countries outside of Europe varies from lower than 3% (Poland) to as high as 25% (Belgium). Standardized assessment of ethnicity is absent amongst most European countries: often proxy measures are used, such as country of birth, and in some high-income countries (HIC) health interview surveys provide most of the population-based data on ethnic minority groups. These data are primarily from Nordic and western European countries^6^ and indicate that variation in CVD rates among immigrants become closer to the average of the host country's citizens with increasing duration of time lived in a given country. Furthermore, CVD rates are strongly influenced by socioeconomic position irrespective of ethnicity. The ESC assessment indicates that HIC in western Europe have the lowest CVD rates compared to countries of any income level in other parts of Europe. Within this western European HIC, CHD rates are higher in men compared to women, whereas stroke rates are approximately equal between the sexes.^48^ GBD 2021 further supports these sex-specific trends and documents consistent patterns in behavioral and metabolic risk factors, such as hypertension, tobacco use, and obesity, which vary by both sex and subregion.^28^

#### Risk factor patterns

The increasing prevalence of obesity in Europe currently constitutes a significant barrier to achieving further reductions in the CVD burden. The GBD 2021 data indicates that obesity now ranks among the top five modifiable risk factors for cardiovascular death and DALYs in nearly all European countries, with higher burdens observed in eastern and southern Europe.^28^ A 2019 European Union (EU) report recorded the highest proportions of obesity (defined as BMI ≥ 30) among women in Estonia (23·6%), Latvia (25·7%), Ireland (26·0%), and Malta (26·7%) and among men in Croatia (23·7%), Ireland (25·7%), Hungary (25·8%), and Malta (30·6%). These obesity proportions are based on national population-level data and are not disaggregated by ethnicity or race because standardized data on ethnicity are not routinely collected in European countries. As a result, while these figures highlight concerning national trends in obesity, they do not provide insight into how obesity may disproportionately affect ethnic minority populations—which in other studies, have been shown to face higher obesity-related risks due to socioeconomic, cultural, and structural factors. Across Europe, the proportion of adults with obesity was lower as the education level rose.^49^ The GBD 2021 analysis confirms the strong inverse association between educational attainment and obesity prevalence, supporting earlier EU findings.^28^ Higher prevalences of hypertension and diabetes between ethnic minority groups compared to white Europeans are commonly reported.^29^ Smoking and physical inactivity are also prevalent cardiovascular risk factors across Europe. Despite the decline in smoking in many European countries, the pace has slowed with the rates plateauing or rising in some countries, particularly among women. Only one in three adults in Europe participates in adequate levels of physical activity, and rates of physical inactivity are typically higher amongst women compared with men, and in high-income compared with middle-income EU countries.^21^

Recent migrants to Europe often face higher CVD risk than the host population.^3^ In the multi-ethnic HELIUS study in the Netherlands, only 30% of ethnic minority participants had BMI 18·5–24·9 kg/m^2^ compared with 60% of the White Dutch population, and perceived ethnic discrimination was significantly associated with higher BMI and waist circumference in the South-Asian Surinamese, African Surinamese, and Turkish groups.^26^ Similarly, the Research on Obesity and Diabetes among African Migrants (RODAM) study compared Ghanaian migrants living in Amsterdam (Netherlands), London (UK), and Berlin (Germany) with Ghanaians residing in urban and rural areas of Ghana, found that Ghanaian migrants had a higher prevalence of CVD than non-migrants.^50^ The CHIP Study (Chinese Immigrant Population Study) in Italy compared first-generation Chinese immigrants with native Italians residing in the same area; Chinese immigrants had a higher prevalence of type 2 diabetes than the native Italians, with a significant proportion of the Chinese immigrants unaware of their glucose status.^27^ Management of diabetes and hypertension, their associated risk factors, and target organ damage has been reported as sub-optimal among ethnic minority groups, including people from Russia or the former Soviet Union, Estonia, and Somalia in Finland, and among people from South Asia, Sub-Saharan Africa, the Caribbean, and Middle East compared with White Europeans in western and northern European health-care settings.^51^^,^^52^ This is partly attributed to differences in SES, dietary habits, and language barriers which can hinder access to health care.

#### Intersection with social disadvantage

Across Europe there is a clear correlation between cardiovascular risk factors of smoking, blood pressure, and obesity and social disadvantage.^48^ Higher levels of education, particularly in women, are associated with lower obesity rates, underscoring the need for targeted interventions by sex, and ideally culturally tailored by ethnic group. Migrants from low-income and middle-income countries in Sub-Saharan Africa, South Asia, and the Caribbean often experience SES decline after they move to Europe, increasing CVD risk due to heightened stress, limited health-care access, and poor living conditions.^3^

#### Health-care system and access

Contextual (e.g., healthcare, systems, politics) and individual (e.g., biology, migration, SES, culture) factors influence the management and prognosis of CVD.^3^ European countries have variable health-care systems: Beveridge-type funding systems provide universal health coverage through general taxation (e.g., in Italy, Spain, and Portugal), whereas in Bismarck-type or taxation systems, mandatory health insurance schemes are financed through employer and employee contributions, and providers are often private, but care is still accessible to all (e.g., in Germany, France, and Belgium).^39^ These system differences contribute to variations in cardiovascular care delivery and outcomes between countries.^39^ While some ethnic groups have higher rates of health-care utilization for specific CVD risk factors (e.g., diabetes care for people of South Asian origin and hypertension care for people of African origin)^53^ compared to Europeans, there is differential health-care access for ethnic minorities within countries.^54^ Some longitudinal and cross-sectional research suggests that, over 20–30 years of residence, cardiovascular risk and health-care use by migrant populations may converge towards that of the majority population.^54^

### United Kingdom

#### Epidemiology of CVD

The UK is a diverse society, with people of color making up approximately 18% of the population. Cardiometabolic risk factors—including high blood pressure, obesity, high cholesterol, diabetes, smoking, and physical inactivity—contribute to an estimated 80% of CVD burden.^31^ Additionally, health inequalities play a significant role, accounting for around 20% of the gap in life expectancy between the most and least deprived communities in England.^31^ The disproportionate burden of cardiometabolic risk factors in more deprived populations—driven by structural determinants like access to healthy food, housing, employment, and healthcare—largely explain this high contribution to unequal health outcomes. The British Heart Foundation estimates that 7·6 million people in the UK have CVD, projected to increase by one million over the next decade given risk factor trends and aging of the population.^31^ Ethnic disparities exist, with Black African, Caribbean, and South Asian populations at higher CVD risk than White counterparts. However, in a Scottish cohort using Census-linked data, the Chinese population had lower cardiovascular mortality rates than the White Scottish population.^55^

#### Risk factor patterns

According to a 20-year population-based cohort study SABRE in the UK, South Asians and African Caribbeans, had a higher baseline prevalence of hypertension and diabetes compared to European individuals. This elevated burden of cardiometabolic risk factors contributes to a higher risk of CHD (subhazard ratio (SHR): 1·70; 95% CI: 1·52, 1·91) and diabetes-associated stroke events (SHR: 2·50; 95% CI: 1·81, 3·45) among South Asians than in the European population. Also from this population-based cohort, African Caribbean people in the UK had the highest diabetes associated stroke risk (SHR: 2·97; 95% CI: 1·83, 4.82), yet a lower risk (SHR: 0·64; 95% CI: 0·52, 0·79) of CHD compared to their European counterparts.^7^ The UK Biobank reinforces the higher CHD risk among South Asians compared to White individuals (HR: 1·24; 99% CI: 1·03, 1·48) even after adjusting for lifestyle and clinical risk factors, whereas Black individuals generally had similar risk to White individuals.^56^ White populations have the highest rates of smoking, while obesity is prevalent across all ethnicities, with South Asian and Black populations more prone to complications such as metabolic syndrome at lower BMI.^57^ These differences are strongly influenced by SES.^58^

#### Intersection with social disadvantage

Health inequities (e.g., unequal access to health care, higher rates of smoking, obesity, diabetes, and poorer living conditions)—account for 20% of the life expectancy gap between the most and least deprived areas in the UK.^59^ The Health Check programme, launched in 2009 in England, was a preventive programme for residents aged 40–74 years designed to assess cardiovascular risk and other risk factors for chronic disease. Health Check showed the major drivers of health inequalities were social disadvantage and deprivation, accounting for the significant variations observed not just between, but within ethnic groups.^30^ Those in the most deprived deciles consistently displayed a higher prevalence of CVD risk factors such as obesity, smoking, physical inactivity and high cholesterol levels and experienced poorer cardiovascular health outcomes.^30^^,^^31^ Compared with the White population, rates of obesity and inactivity were higher in South Asian and Black populations, particularly women.^30^ Conversely risk of smoking and alcohol use was lower among people from minority ethnic groups than in White individuals.^30^

#### Health-care system and access

The UK has universal health-care access with well-developed social and primary care, but disparities exist in cardiovascular treatment. South Asian and Black populations receive fewer interventions for heart disease, such as angioplasty and bypass surgeries, despite presenting with more severe conditions.^40^^,^^41^ Undocumented migrants and asylum seekers face greater obstacles in accessing care, delaying diagnosis and treatment due to their concern that seeking medical attention—particularly at government-affiliated institutions—could lead to their personal information being shared with immigration authorities and result in detention or deportation.^60^ Structural racism discrimination and language barriers further limit access for ethnic minority groups.^61^^,^^62^ The Health Check program assessed more than 9·5 million people but was not a random sample of the population and had selection biases, although it provides some useful insights into cardiovascular risk factor levels by age, ethnicity, and SES. Attendance at Health Checks was consistently lower in more deprived areas, regardless of ethnicity. However, individuals from Black, South Asian, and other minority ethnic groups were more likely than White individuals^30^ to receive appropriate follow-up care and adhere to recommended interventions once they did attend.^30^

After nine years of follow-up, those who attended the Health Checks had significantly fewer CVD admissions and lower rates of cardiovascular and all-cause mortality compared to non-attendees.^63^

### USA

#### Epidemiology of CVD

CVD claims over 900,000 lives in the USA annually, adding inordinate strain on individuals, health-care systems, and health expenditures.^44^ Racial and ethnic minorities constitute approximately 40% of the US population and bear a disproportionate burden of adverse health outcomes. Black Americans, American Indians, and Hispanic populations experience significantly higher rates of cardiovascular risk factors than White populations.^16^ Black adults are more than twice as likely to die from CVD as White adults, and American Indians individuals also face a higher prevalence of CHD. Black American women and men bear a disproportionately higher burden of CVD risk factors, CVD, and its adverse outcomes compared to White counterparts.^22^ The Asian population, the most rapidly growing minority ethnic population in the USA, is not only under-studied, but varies widely in country of origin, such that considering a homogenous Asian group fails to recognize the heterogeneity in risk of CVD observed, and ethnicity should be examined. For example, South Asian Americans have some of the highest rates of diabetes and the highest CVD rates, whereas Chinese Americans have some of the lowest rates for both.^9^ Reasons for these intra-Asian differences are likely attributable to a complex array of factors including immigration patterns, socioeconomic differences, acculturation, health literacy, and for some risk factors differences in genetic variant frequencies.^64^

#### Risk factor patterns

The prevalence of traditional CVD risk factors varies broadly across race groups in the USA, with significant gradients from low to high going from North-South, West to East, and socioeconomic position. For example, daily smoking is more prevalent among White and Black adults (both 11·7%, compared to Asian (5·4%) and Hispanic (7·7%) adults) and in those with low-incomes (18·3%), compared to high-income (6·7%).^44^ Obesity disparities are also evident by sex and race, with Black females having the highest prevalence (57·9%) of obesity.^44^ Analysis of the 2013–2017 National Health Interview Survey found a positive association between obesity prevalence with higher cumulative social determinants of health—measured across 38 indicators and grouped into quartiles, with the greatest differences in obesity rates observed between people with most vs. fewest negative social determinants of health indicators, most notably among non-Hispanic Black adults, especially women.^32^ Black, American Indian, and Hispanic Americans have a higher prevalence of traditional cardiovascular risk factors compared to White Americans, and South Asians have both a greater likelihood and a higher number of cardiometabolic risk factors, including type 2 diabetes. This burden, of cardiometabolic risk factors, is highly context dependent, being influenced by geography, education, and SES.^32^

Psychosocial stress, often stemming from economic insecurity, discrimination, and poor living conditions, activates stress-related biological responses and exacerbates hypertension, diabetes, and other risk factors, disproportionately affecting minority ethnic populations.^3^^,^^65^

#### Intersection with social disadvantage

In the USA, people with higher income have lower morbidity and mortality from CVD.^66^ While life expectancy has consistently risen among high-income individuals, it has not in the remaining population.^67^ Higher earners tend to be more highly educated, more likely to be married, have more social support,^68^ have better access to health care, be more able to afford medications, have better housing, consume healthier food, and live in less polluted and safer neighborhoods. Longitudinal associations have shown greater upward economic mobility is associated with lower CVD risk and mortality.^69^ Low educational attainment leads to poorer employment prospects, economic uncertainty, food insecurity and poor health literacy that ultimately contributes to unhealthy behaviors, medication non-adherence, provider-patient communication barriers and impaired healthcare access, often because of inadequate insurance, all factors that are strongly associated with CVD risk factors and CVD outcomes.^70^ Recent reports from the American Heart Association have emphasized the crucial role of the social determinants of health, including structural racism, in determining risk of CVD in underserved minorities, particularly for the Black and American Indian populations.^22^^,^^45^

#### Health-care system and access

Americans from minority ethnic or racial backgrounds, particularly those of lower SES, face greater barriers in accessing health care and also experience lower quality of care when they do access it compared with White Americans.^71^ Black and Hispanic people are two-to-four times less likely to have health insurance than White individuals.^72^ More people from racial and ethnic minorities experience delayed access to health care for cost reasons compared to White Americans, thus delaying treatment and increasing the likelihood of developing advanced CVD.^16^ Black and Hispanic patients, especially women, receive fewer interventions, such as coronary artery bypass or timely treatments after myocardial infarction, compared to White patients.^9^ These disparities contribute to worse outcomes, including higher hospital readmission rates and mortality, and are often linked to systemic biases within health-care systems.^16^

### Canada

#### Epidemiology of CVD

Canada is a multiethnic, multicultural nation, with 26·5% of the country made up of ethnic minority populations.^10^ While South Asians and Chinese are the two largest non-White ethnic groups in Canada, recent immigration patterns to Canada include people from the Middle East, Syria, Afghanistan, and Ukraine. The history of Black communities in Canada spans over 400 years, beginning as enslaved people with a wave of migration during the American Revolution, and more recently through immigration from African and Caribbean nations.

CVD remains a leading cause of morbidity and mortality in Canada. The risk of CVD varies across different populations, reflecting a complex interplay of ethnicity, country of origin, SES, and gender.^36^^,^^73^ The age-standardized 10-year incidence of major cardiovascular events—defined as acute myocardial infarction, stroke, coronary revascularization, or cardiovascular death—varied substantially, with East Asian male and female immigrants having the lowest risk (2·4 and 1·1 per 1000 person-years, respectively) and South Asian immigrants having the highest risk (8·9 and 3·6 per 1000 person-years, respectively). Although the incidence of major cardiovascular events among East Asian, Black, and Southeast Asian individuals increased with longer duration of residence in Canada, it did not increase among South Asians.^36^ Possible contributing factors requiring additional research include: 1) variations in dietary practices, alcohol intake, and physical activity levels; 2) pre-migration environmental exposures (e.g., urbanization, air pollution, exposure to second hand smoke, health care access); 3) accuracy in measuring certain risk factors (e.g., blood pressure, incomplete smoking and lipid data); 4) cultural differences in health-seeking practices; 5) variations in the proportion and characteristics of immigrants who decide to return to their home country; and 6) genetic variants that differ in frequency between ethnic groups.^34^, ^35^, ^36^

#### Risk factor patterns

South Asians represent the largest non-White ethnic group in Canada and have a significantly higher prevalence of diabetes, metabolic syndrome, and gestational diabetes than White Canadians.^34^^,^^35^ Black Canadians exhibit higher rates of hypertension than White Canadians, Black women have higher rates of obesity and type 2 diabetes than other groups, and Chinese Canadians, typically have lower rates of obesity and CVD than White Canadians, but with weight gain, are at risk of developing the metabolic syndrome.^33^

#### Intersection with social disadvantage

Socioeconomic factors play a significant role in CVD risk across ethnic groups in Canada, and those with higher social disadvantage have a greater burden of CV risk factors.^2^ In a population-based study, socially disadvantaged Indigenous women and South Asian women living in Canada had a higher predicted probability of CVD compared with Canadians of European descent and Chinese men living in Canada.^2^ Immigration status influences the relationship between neighborhood level income and cardiovascular events, with clear income-related decline in event risk among long-term residents and less pronounced patterns among more recent immigrants (e.g., immigrated after 1985).^74^

#### Health-care system and access

Despite Canada's universal healthcare system, minority ethnic populations and recent immigrants face barriers to accessing health care, contributing to disparities in CVD outcomes. Low SES, language barriers, and inadequate health-care infrastructure in underserved neighborhoods limit access to proper diagnosis and treatment. Historically underserved racial and ethnic populations experience delays in receiving care, lower utilization of health services, and an inability to afford and access prescription medications, requiring systemic strategies.^42^ Immigrants—particularly refugees, temporary migrants, and international students—are more vulnerable to health-care inequities, with female immigrants and those with disabilities being disproportionately affected.^75^

### Mexico, Central America, and the Caribbean

#### Epidemiology of CVD

CVD is the leading cause of mortality and burden of disease and disability in Mexico, Central America, and the Caribbean.^47^ Ethnic diversity in Mexico, Central America, and the Caribbean stems from colonization, and standardized data on ethnicity, race or Indigeneity are not routinely collected, which limits clear data on CVD patterns and risk factor trends. People of mixed European, African, and Indigenous ancestry (referred to as Mestizaje [the process of mixing] or Mestizo [a person of mixed heritage]) make up an estimated 62% of the population of Mexico; 21% self-identify predominantly as Indigenous, and 10% as mostly European.^20^ In Mexico, CHD is the leading cause of CVD-related deaths, though rates declined between 1990 and 2017. Central American countries (Belize, Costa Rica, El Salvador, Guatemala, Honduras, Nicaragua, and Panama) face a dual burden of CVD and communicable diseases. The Caribbean including Trinidad and Tobago and Guyana, have a diverse population of African, South Asian, Indigenous, European, Chinese, and Portuguese descent. Collection of demographic data is improving in the Caribbean through CARICOM, an organization of 15 Caribbean nations which aims to improve social, economic, and health outcomes across the region which has specific health and social equity initiatives.^11^ The Caribbean has the highest CVD mortality rates compared to Mexico and Central America in this region, with CHD and stroke as the leading causes of death. Data from the PAHO ENLACE dataset reporting CVD mortality rates per 100,000 population for year 2019 shows that the Non-Latin Caribbean (e.g., Jamaica, Trinidad, and Tobago) stand out with the highest CVD mortality rates (196·7 per 100,000), exceeding rates Central America, Mexico, and the Latin Caribbean (e.g., Haiti and Cuba) at 169·6.^23^ South Asians in Guyana and Trinidad experience higher CHD rates than the rest of the population, while Afro-descendant populations face a greater burden of stroke, largely attributed to hypertension.^24^ While GBD 2021 offers valuable information on the overall CVD burden and associated risk factors in the Caribbean, Central America, and Mexico, the absence of ethnicity-specific data highlights the need for more targeted research.^28^

#### Risk factor patterns

Across the Caribbean, Mexico, and Central America, over 40% of adults are obese, 45% are hypertensive, and 25% have diabetes in some ethnic groups. Compared to White individuals in the region, Afro-Caribbean adults in the Caribbean have very high rates of hypertension, moderate to high rates of diabetes, and high obesity levels, especially among women. South Asian populations have high rates of diabetes, along with high rates of hypertension and moderate obesity. Mestizo populations, especially in Mexico and Central America, have very high rates of diabetes and obesity, and moderate to high rates of hypertension.^37^

#### Intersection with social disadvantage

Social disadvantage, such as poverty and limited access to healthy food, contributes to rising obesity and CVD risk in all countries in this region. Even in countries where there has been more equal access to healthcare such as Costa Rica, CVD mortality remains higher among disadvantaged groups, which disproportionately affects non-White ethnic groups.^47^

#### Health-care systems and access

In Mexico, access to health care is depends on labor status and the ability to pay. Individuals with a greater cardiovascular risk factor burden are more likely to be lower income and have reduced access to health services. In Central America and the Caribbean in 2019, 37% of people with hypertension were undiagnosed, 15% diagnosed were untreated, and 47% treated did not achieve controlled blood pressure (<140/90 mm Hg)^38^ and hypertension more common amongst the poor. These factors disproportionately affect ethnic groups such as Afro-Caribbean, Indigenous, and other non-White populations. This difference is important to demonstrate as optimal screening and treatment could avert substantial deaths from CHD and stroke.^76^ In Costa Rica, the decline in CVD is attributable to health system reform focused on primary prevention strategies that can serve as a model for other nations in this region. Costa Rica's health gradient is modest given the buffer effect of its universal healthcare system which covers 90% of people in the country.^43^

### Regional patterns of CVD and cardiovascular risk experienced by Indigenous Peoples

#### Epidemiology of CVD

Worldwide, there are over 476 million Indigenous people, accounting for 6·2% of the global population.^5^ Indigenous peoples have historical connections to regions that have existed before colonization. While Indigenous peoples around the world have distinct ancestry, languages and cultural practices, they share similar colonial histories, which have resulted in marginalization and acculturation. Colonization has disrupted Indigenous peoples' traditional ways of living and has had a major impact on their overall and CV health. There are limited contemporary, high-quality CVD data, and what data are available are further limited by misclassification of ancestry,^45^ small sample sizes, and possible underreporting or misreporting of death and disease in Indigenous populations.^77^ The scarcity of high-quality data is especially apparent among less populous Indigenous groups (i.e., Métis and Inuit of Canada, Indigenous Peoples of Russia, and Native Hawaiians). Despite gaps in contemporary data, herein we report the CVD and risk factor burden in all Indigenous peoples in Europe and North America for whom data are available (Appendix S2).

In Europe, the most populous Indigenous populations include the Sámi of Northern Scandinavia, the Greenlandic Inuit and 40 officially recognized Indigenous Peoples in Russia. While there is limited data regarding CVD among Russia's Indigenous populations are scarce, the Sámi and Greenlandic Inuit both historically had lower rates of CVD than their respective non-Indigenous populations; however, over the past two decades, higher incidences of CHD and stroke have since been reported in the Sámi and Greenlandic Inuit, though data in the last 5 years is limited.^13^, ^14^, ^15^

The American Indian/Alaska Native (AI/AN) population comprises approximately 1% of the USA, and has the highest prevalence of CHD compared to other race-classified groups in the USA, with earlier age of onset.^17^^,^^78^ Decline in CHD prevalence was also slower in AI/AN from 2000 to 2018.^17^^,^^77^^,^^79^^,^^80^ In the USA, Native Hawaiian also have higher rates of CVD than White individuals in the USA.^18^ Settler colonialism, government relocation programmes, and intergenerational trauma have contributed significantly to the disparate health outcomes of the Indigenous population in the USA.^80^, ^81^, ^82^ Although past treaties have improved access to federal health-care services, distance from specialized cardiovascular care is often a barrier to access.^45^^,^^83^

In Canada, Indigenous peoples (First Nations, Inuit, and Métis) make up approximately 5% of the population.^19^ Colonization in Canada, including loss of traditional lands, assimilationist policies and intergenerational trauma incurred through the Residential School system, have had major impacts on Indigenous Canadians' health and wellness, including cardiovascular health.^46^^,^^84^ While rates of heart disease were historically lower in First Nations individuals compared to the non-Indigenous population, they now experience significantly higher rates of CVD.^85^, ^86^, ^87^, ^88^ Compared with non-Indigenous Canadians, Métis people also face higher rates of heart disease, including heart failure, stroke, and acute coronary syndrome.^89^ CVD data from Canadian Inuit is limited but suggest higher rates of myocardial infarction and stroke than the general population.^90^

Scarce data exist on Indigenous peoples' cardiovascular health in Mexico and Central America as there is substantial admixture with European colonizers, and ethnicity is not measured in a standardized way.^25^ In several of these countries, governmental policies have promoted the acculturation of Indigenous peoples to varying degrees, in turn increasing cardiovascular risk factors among some Indigenous communities.^91^^,^^92^ Conversely, Indigenous groups maintaining traditional lifestyles tend to have lower rates of CVD, as seen in the Tepehuan people and island-dwelling Kuna, who have lower hypertension rates than their urban counterparts. Considering the diversity within and among Indigenous communities, there are shared common structural factors that contribute to CVD disparities including land loss and environmental degradation due to colonialism, underfunded healthcare systems, and significant socioeconomic marginalization.^93^

#### Intersection with social disadvantage

The intergenerational legacy of colonization and acculturation are observed amongst all Indigenous peoples irrespective of region. This has drastically hindered the ability for Indigenous people to maintain their traditional diets and active lifestyles, contributing to higher rates of metabolic syndrome and CVD.^91^^,^^92^ For Indigenous people, higher rates of cardiovascular risk factors are directly linked to the legacy effects of colonialism, underpinning the persistent socioeconomic disadvantage they face, including poverty, food insecurity, poor housing, and limited access to health care.^46^^,^^94^^,^^95^ This is compounded by psychological stressors and increased substance use rates in some Indigenous people, rooted in the intergenerational trauma from colonization.^45^^,^^46^^,^^87^^,^^96^

Indigenous women are particularly vulnerable to the compounding effects of social and cultural factors, which leads to higher rates of cardiovascular health disparities compared to Indigenous men.^78^^,^^97^ Sex and gender-based disparities in cardiovascular health outcomes have been observed in Indigenous peoples in Europe, the USA, and Canada. For example, Sámi women, but not men, have been shown to have higher rates of cardiovascular mortality compared to non-Sámi counterparts, with Sámi women having slower rates of decline in cardiovascular risk compared to non-Sámi women.^98^ Similarly in the USA, AI/AN women have had an increase in proportionate mortality from premature MI over the last 20 years, while rates decline in AI/AN men.^78^ In Canada, First Nations and Inuit women have been found to have higher rates of cardiovascular mortality and MI, respectively, compared to the non-Indigenous Canadian population.^90^^,^^97^

To reverse the adverse effects of colonization, Indigenous peoples in many countries are reclaiming their rights to self-determination. Social factors, such as trust in the medical community, social support networks, and access to higher education and affordable medications can lead to improvements in cardiovascular health outcomes in Indigenous communities.^46^

#### Health care systems and access

Inadequate access to health insurance has been linked to worse cardiovascular outcomes in Indigenous populations in the USA and Mexico.^99^^,^^100^ Indigenous populations face other geographic and infrastructural barriers to both primary care and specialized CV care (including cardiovascular diagnostics, percutaneous intervention and cardiac surgery)^77^^,^^95^; and those with limited access face higher cardiovascular risk.^46^ In Indigenous communities, self-governance, improved access to primary care and acceptance of traditional Indigenous ways of knowing has been strongly associated with improved control of cardiovascular risk factors.^101^^,^^102^

## Conclusions

Despite persistent misconceptions that race is biological, we advocate for the safe, stigma-free collection of self-reported ethnicity, ancestry, and Indigeneity data in health research. These data help identify high-risk communities,^27^ and intersecting socioeconomic factors, and can drive action to close the health equity gap (Panel 2). For example in the USA, women from minority ethnic backgrounds facing social disadvantage struggle most with health-care access, experience higher rates of undiagnosed CVD, and have worse cardiovascular outcomes.^106^

Panel 2 **Overall and region specific recommendations.**

**Table:** 

**Actions**	
**Overall**	Implementation of preventive measures: • Ensure effective and high-quality preventive and management measures. • Address disparities regardless of gender, sex, geographical location, SES, or ethnic composition. Culturally-tailored guidelines: • Adapt diagnostic and treatment guidelines to reflect cultural diversity. • Ensure equitable access and trust in the health-care system for migrants. Training for health-care workers: • Educate health-care workers on cultural nuances affecting health behaviors. • Consider variations in food patterns, smoking habits, and physical activity. Targeted prevention programs: • Address increasing rates of overweight, obesity, hypertension, and diabetes among minority ethnic groups influenced by SES. • Develop tailored prevention programs for specific ethnic groups.^103^^,^^104^ Ethnic-specific health policies: • Implement programs for high-risk groups, such as South Asians, to address premature CHD. • Establish educational programs for physicians and citizens, along with specialized screening initiatives. Role of local health-care providers: • Emphasize the importance of culturally sensitive education and management programs. • Adapt approaches based on local cultural and societal gender norms and socioeconomic factors.
**Regional considerations**	
Europe	Targeted public health initiatives: • Develop screening and prevention programs similar to the NHS Health Check to identify and manage CV risk factors early among migrant and ethnic minority populations. • Focus interventions on high-risk groups, including migrants from South Asia, Sub-Saharan Africa, and the Caribbean, and low-SES communities. Primary care for vulnerable populations: • Expand access to culturally competent primary care services for recent migrants, refugees, asylum seekers, and undocumented populations across European health systems. Culturally appropriate healthcare services: • Integrate culturally and linguistically tailored services to improve healthcare engagement among diverse populations. • Promote health literacy initiatives targeted at ethnic minority and migrant communities to better manage CV risk factors. Reducing stigma: • Implement public health campaigns to reduce stigma surrounding chronic diseases, particularly in migrant and ethnic minority communities, to encourage early diagnosis and management. Addressing data gaps: • Improve standardized data collection on ethnicity, migration status, and social determinants of health across Europe. • Prioritize inclusion of migrants and ethnic minorities in clinical trials and population health research to better inform policies and interventions aimed at reducing CV disparities.
UK	Targeted public health initiatives: • Implementing programs like the NHS Health Check to detect and manage risk factors early. • Focus on high-risk groups, including South Asians and individuals in deprived areas.^69^ Primary care for vulnerable populations: • NHS provides care for refugees, asylum seekers, and those who have been refused asylum. Culturally appropriate healthcare services: • Integrate services that respect cultural sensitivities. • Improve health literacy within ethnic minority communities. Reducing stigma: • Efforts to reduce stigma surrounding chronic diseases in ethnic minority populations. Addressing data gaps: • Limited research on smaller ethnic groups and underrepresentation in clinical trials. • Strengthen data collection and involving minority communities in health research are essential for reducing disparities and improving CV outcomes.
United States	Targeted public health initiatives: • Develop national programs modeled on successful preventive strategies to detect and manage CV risk factors early in racial and ethnic minority populations, with a focus on Black, American Indian, Hispanic, and South Asian communities. • Address psychosocial stressors such as economic insecurity and discrimination through integrated public health interventions that promote protective factors like social support networks. Primary care for vulnerable populations: • Expand access to primary care and preventive services for uninsured and underinsured individuals, particularly targeting Black, Hispanic, and American Indian communities. • Strengthen community health programs to deliver culturally tailored services closer to where underserved populations live. Culturally appropriate healthcare services: • Promote healthcare delivery models that recognize the heterogeneity within racial categories, particularly among Asian Americans, and tailor prevention and treatment approaches accordingly. • Improve health literacy, food security, and medication access in racial and ethnic minority communities to reduce disparities in CV outcomes. Reducing stigma: • Launch community-driven campaigns to reduce stigma surrounding chronic diseases and healthcare engagement, particularly within Black, Hispanic, American Indian, and Asian American communities. Addressing data gaps: • Strengthen the disaggregation of race and ethnicity data in health research to accurately capture the diverse risk profiles within racial groups (e.g., South Asian vs. Chinese Americans). • Prioritize the inclusion of racial and ethnic minorities in clinical trials and longitudinal studies to better understand and address disparities in CV risk and outcomes.
Canada	Culturally appropriate healthcare services: • Recognize the diverse health needs stemming from Canada's complex immigration history and multicultural identity. Ethnic-specific risk factors: • Acknowledge higher rates of diabetes among South Asian and Indigenous people. • Address elevated hypertension rates among Black Canadians. • Implement tailored healthcare approaches to meet these specific needs. Environmental factors: • Consider the impact of poor neighborhood conditions and rising costs of living. • Address food insecurity, which exacerbates CVD risks for marginalized populations. Strategies for improvement: • Enhance data collection to better understand health disparities. • Utilize intersectional approaches to address diverse health challenges. • Promote culturally safe healthcare practices to reduce health inequities and improve CVD outcomes.
Central America	Policy initiatives: • Mexico's taxation of sugar-sweetened beverages (SSBs) aims to reduce obesity and diabetes, especially in lower-income groups. Critical efforts in Latin America and the Caribbean: • Improve hypertension care and strengthen health systems to reduce health inequities. • Design and implement screening programs and low-cost treatment options based on WHO Best Buys. Successful models: • Costa Rica's primary prevention model has proven effective in reducing CVD, offering valuable lessons for other nations.
Indigenous populations	Addressing environmental impacts on cardiovascular health: • Acknowledge the cardiovascular (CV) impacts of industrial activity (e.g., oil pollution) on Indigenous lands, and take action to remediate environmental harm that contributes to CV health disparities (i.e., exposure to heavy metals and organic pollutants in the Canadian Inuit and exposure to arsenic and cadmium from groundwater contamination in AI/AN communities). • Support prevention and treatment efforts for CAD, PAD, and stroke, which are more prevalent due to these environmental exposures. Improving data systems and equity in research: • Invest in collecting high-quality, contemporary data on cardiovascular disease in Indigenous populations, led and governed by Indigenous communities. • Address misclassification of ancestry, small sample sizes, and health outcome misreporting by reforming data collection practices with cultural and contextual appropriateness. • Close data gaps in structural heart disease, arrhythmias, and heart failure—particularly for less populous Indigenous groups (e.g., Métis, Inuit, Indigenous Peoples of Russia, Native Hawaiians)—through inclusive research funding and infrastructure. Ensuring representation in genomic research: • Prioritize the ethical inclusion of Indigenous Peoples in genetic/genomic studies to ensure equitable access to emerging precision medicine and to redress long-standing exclusion. Supporting Indigenous sovereignty and self-determination: • Recognize and support Indigenous Peoples' reclamation of self-determination as foundational to improving health outcomes and reversing the harms of colonization. • Empower Indigenous leadership in health governance, policy development, and program delivery. Addressing social determinants of cardiovascular health: • Build trust in the medical system by investing in culturally safe care and Indigenous-led health services. • Strengthen social support networks, access to education, and availability of affordable medications—all crucial for cardiovascular health equity in Indigenous communities.^105^

Social disadvantage can outweigh protective features of race and ethnicity (e.g., in White populations) or compound risks (e.g., Black and Indigenous peoples). Alongside gender and race/ethnicity, structural factors such as racism should be recognized as key drivers of health disparities, reinforcing systemic inequalities that impede progress toward health equity.

While some people advocate for removing race and ethnicity from clinical history, we put forth that their inclusion remains crucial for addressing disparities in CVD outcomes and guiding patient care; the benefits of acknowledging these factors outweigh the risks of stereotyping.^107^ Although there is a legitimate concern that misuse or overreliance on ethnic categories could lead to stereotyping, misdiagnosis, or undertreatment, the risks can be mitigated through careful, context-specific interpretation. For example, Black people with hypertension may require targeted stroke prevention, South Asians with atypical chest pain need CHD assessment, and Indigenous patients may benefit from lifestyle-based glucose management. Physician awareness of these patterns enhances diagnosis and treatment. Culturally tailored dietary discussion and language-concordant care improve patient outcomes.^108^ Ignoring racial and ethnic differences overlooks their social impact. Recognizing these differences is key to culturally sensitive care and personalized medicine, ensuring prevention and treatment aligns with patient needs.^109^

Beyond collecting ethnicity data, governments and industry must ensure research reflects affected populations, at a minimum aligning with Census proportions. Increasing ethnic diversity in clinical research builds trust, enhances adherence to evidence-based treatments, and improves access to healthcare services. This improved access leads to better quality of life and overall health status. Consequently, reducing health disparities through such inclusive research practices results in significant healthcare cost savings.^110^

**Fig. 2:**
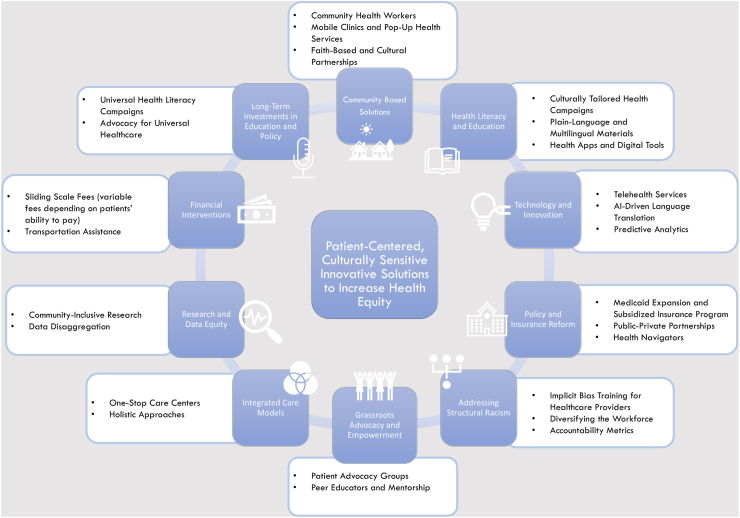
**Innovative solutions to improve health disparities.** Figure presents patient-centered, culturally sensitive solutions aimed at increasing health equity. Blue boxes represent broad solution categories; white boxes outline specific interventions within each category. While all proposed interventions aim to reduce health disparities—including cardiovascular disease—some may be more impactful depending on context. The basis for these interventions stems from expert recommendations, policy initiatives, and published consensus guidelines.

Other strategies to optimize health equity include provision of universal healthcare to eliminate financial barriers, paired with robust social programmes addressing health determinants such as housing, education, and employment. Culturally adapted health literacy initiatives and language services, and community-focussed approaches such as community health workers, can bridge gaps for marginalized communities, while investing in preventive care and primary health-care accessibility for high risk can facilitate early intervention. Tackling structural inequities through policies against ethnic discrimination, and workforce diversity are crucial, as is incorporating data-driven decision-making to identify and address disparities (Fig. 2). Several countries have made significant progress in reducing health equity gaps, such as Norway, Rwanda, and Costa Rica.^43^

In summary, the safe collection of self-reported ethnicity data can inform clinical decision making and broaden the generalizability of clinical research.^1^ Collecting such information enables high risk communities to be prioritized for access to health care and research and will build trust between these communities and public health providers, all of which is required to deliver evidence-proven therapies to prevent and treat CVD. Second, taking an intersectional perspective is crucial as ethnicity strongly intersects with social disadvantage and gender, and combine to powerfully influence cardiovascular health. While optimizing social and economic factors can lead to improved health, these socio-political changes are often outside the reach of influence for practicing clinicians. Advocacy of health-care workers for marginalized communities, improvements in health literacy, cultural tailoring of health messaging strategies, together with policies focussed on health of immigrants, can lead to more equitable health-care access for those who have cardiovascular risk factors and CVD, and can lead to tangible improvements in the health of marginalized populations.

## Contributors

SSA, SK, and MM contributed to the conceptualization, data curation, formal analysis, investigation, methodology, writing of the original draft, and review and editing of the manuscript. MG and AAQ contributed to the conceptualization, writing of the original draft, and review and editing of the manuscript. MK and JD contributed to the writing of the original draft and manuscript review and editing.

## Declaration of interests

SSA holds the Heart and Stroke Foundation Michael G DeGroote Chair in Population Health, a Canada Research Chair in Ethnic Diversity and Cardiovascular Disease. Dr. Anand has received speaking fees from Novartis, Novo Nordisk, and Amgen in the past year. MM is the recipient of the Canadian Cardiovascular Society/Pfizer/Canadian Heart Function Alliance Research Fellowship in First Nations, Inuit, and Métis Communities Experiencing Heart Failure Inequities. MM has received speaking honoraria from Bristol Myers Squibb. MG is supported by contracts from the National Heart, Lung, and Blood Institutes nos. N01-HV-068161, N01-HV-068162, N01-HV-068163, N01-HV-068164, grants U01 HL064829, U01 HL649141, U01 HL649241, K23 HL105787, K23 HL125941, K23 HL127262, K23HL151867, T32 HL069751, R01 HL090957, R03 AG032631, R01 HL146158, R01 HL146158-04S1, R01 HL124649, R01 HL153500, U54 AG065141, General Clinical Research Center grant MO1-RR00425 from the National Center for Research Resources, the National Center for Advancing Translational Sciences Grant UL1TR000124, Department of Defense grant PR161603 (CDMRP-DoD), and grants from the Gustavus and Louis Pfeiffer Research Foundation, Danville, NJ, The Women's Guild of Cedars-Sinai Medical Center, Los Angeles, CA, The Ladies Hospital Aid Society of Western Pennsylvania, Pittsburgh, PA, and QMED, Inc., Laurence Harbor, NJ, the Edythe L. Broad and the Constance Austin Women's Heart Research Fellowships, Cedars-Sinai Medical Center, Los Angeles, CA, the Barbra Streisand Women's Cardiovascular Research and Education Program, Cedars-Sinai Medical Center, Los Angeles, CA, The Society for Women's Health Research, Washington, D.C., the Linda Joy Pollin Women's Heart Health Program, the Erika Glazer Women's Heart Health Project, the Adelson Family Foundation, Cedars-Sinai Medical Center, Los Angeles, CA, Robert NA. Winn Diversity in Clinical Trials Career Development Award (Winn CDA) and the Anita Dann Friedman Endowment in Women's Cardiovascular Medicine & Research. This work is solely the responsibility of the authors and does not necessarily represent the official views of the National Heart, Lung, and Blood Institute, the National Institutes of Health, or the U.S. Department of Health and Human Services. Consultant Fees/Honoraria: Esperion, Medtronic Inc, unrelated to this work. JD has received CME honoraria and/or consulting fees from Aegerion, Amgen, Astrazeneca, Bayer, Boehringer Ingelheim, Merck, Novartis, Novo Nordisk, Pfizer, Sanofi, Takeda. Research grants from British Heart Foundation, MRC(UK), NIHR, PHE, MSD, Pfizer, Aegerion, Colgate, Roche. He is a member of the Study Steering Committees for Novo Nordisk (SOUL and SELECT). AAQ has been supported by NIH grants P01HL154996-01A1, R33HL138657-05, 5T32 HL130025, P30DK111024-07S2, R01HL166004-01, 5R01HL158141-043, 3R01HL157311-03S1, 1R01HL166004-01.

## Appendix A Supplementary data

Appendices S1 and S2
