# Supplementary material for: Reducing inequalities in cardiovascular disease: focus on marginalized populations considering ethnicity and race
Source: Lancet Reg Health Eur. 2025 Aug 21;56:101371. doi: 10.1016/j.lanepe.2025.101371 (PMC12587327; doi:10.1016/j.lanepe.2025.101371)
Supplement: Appendices S1 and S2 [file mmc1.pdf]

Supplemental Data

Table of Contents

Appendix 1. Search strategy 2

Appendix 2. Indigenous Supplemental Data 3

References 4

Appendix 1. Search strategy

We conducted a structured MEDLINE search via OVID from database inception to July 21, 2024. A top-up search was conducted on April 4, 2025 to capture any updates. We supplemented this search with a hand search of reference lists of included studies, review of subsequent publications related to parent studies, and expert review.

The following terms were paired with regional terms: Ethnicity/ OR ethnic*.ti,ab,kf,kw OR Racial Groups/ OR racial*.ti,ab,kf,kw. AND exp Cardiovascular Diseases/ OR ((cardiovascular or cardio-vascular or heart or coronary) adj2 disease*).ti,ab,kf,kw. AND exp Diagnosis/ OR diagnos*.ti,ab,kf,kw. OR exp Therapeutics/ OR treatment*.ti,ab,kf,kw. OR therapeutic*.ti,ab,kf,kw. OR exp Mass Screening/ OR screen*.ti,ab,kf,kw. AND limit to (english language)

EASTERN EUROPE: europe, eastern/ or bulgaria/ or czech republic/ or hungary/ or moldova/ or poland/ or romania/ or russia/ or slovakia/ or ukraine/ or "Republic of Belarus"/ OR (Belarus or Bulgaria or Czechia or Hungary or Poland or Moldova or Romania or Russia* or Slovakia or Ukraine).ti,ab,kf,kw. OR eastern europe*.ti,ab,kf,kw

WESTERN EUROPE: western europe*.ti,ab,kf,kw. OR (AustriaOR Belgium or France or Germany or Liechtenstein or Luxembourg or Monaco or Netherlands or Switzerland).ti,ab,kf,kw. OR austria/ or belgium/ or france/ or germany/ or liechtenstein/ or luxembourg/ or monaco/ or netherlands/ or switzerland/

UK: united kingdom/ or channel islands/ or guernsey/ or england/ or northern ireland/ or scotland/ or wales/ OR (United Kingdom or Great Britain or Northern Ireland or Wales or Scotland or UK or Channel Islands or Guernsey).ti,ab,kf,kw.

CENTRAL AMERICA: central america/ or belize/ or costa rica/ or el salvador/ or guatemala/ or honduras/ or nicaragua/ or panama/ or mexico/ OR (Central America or Belize or Costa Rica or El Salvador or Guatemala or Honduras or Mexico or Nicaragua or Panama).ti,ab,kf,kw.

CARIBBEAN ISLANDS: caribbean region/ or aruba/ or caribbean netherlands/ or curacao/ or sint maarten/ or west indies/ or "antigua and barbuda"/ or bahamas/ or barbados/ or british virgin islands/ or cuba/ or dominica/ or dominican republic/ or grenada/ or guadeloupe/ or haiti/ or jamaica/ or martinique/ or puerto rico/ or "saint kitts and nevis"/ or saint lucia/ or "saint vincent and the grenadines"/ or "trinidad and tobago"/ or united states virgin islands/ OR (Caribbean or Anguilla or "Antigua and Barbuda" or Aruba or Bahamas or Barbados or "Bonaire, Sint Eustatius and Saba" or British Virgin Islands or Cayman Islands or Cuba or Curacao or Dominica or Dominican Republic or Grenada or Guadeloupe or Haiti or Jamaica or Martinique or Montserrat or Puerto Rico or Saint Barthelemy or "Saint Kitts and Nevis" or Saint Lucia or Saint Martin or "Saint Vincent and the Grenadines" or Sint Maarten or "Trinidad and Tobago" or "Turks and Caicos Islands" or United States Virgin Islands or West Indies).ti,ab,kf,kw. OR (Netherlands Antilles or Greater Antilles or Lesser Antilles).ti,ab,kf,kw.

CANADA: Canada/ OR Canada*.ti,ab,kf,kw.

USA: United States/ OR United States.ti,ab,kf,kw. OR USA.ti,ab,kf,kw.

INDIGENOUS ANCESTRY: Filters applied from all of the provinces of Canada, Alaska, USA, Mexico, Central America, Northern Europe, Greenland and Russia from the following University of Alberta search filters: https://guides.library.ualberta.ca/health-sciences-search-filters/indigenous-peoples.

Appendix 2. Indigenous Supplemental Data

Europe

The Sámi are Indigenous people of Northern Scandinavia, predominantly located in Norway, whose traditional lifestyles involved reindeer herding and fishing.[1] Historically, the Sámi had lower cardiovascular mortality than the general population. However, recent studies show no significant differences in heart disease rates but higher incidence of stroke in the Sámi population (HR 1·36, 95% CI 1·10-1·68).[2] Greenland became a Danish territory in 1814, and the Greenlandic Inuit were initially observed to have lower rates of coronary heart disease, although more recent evidence shows a 2·5 times higher risk of stroke mortality in Greenland compared to Denmark, with similar rates of coronary heart disease observed.[3,4] In Russia, there are 40 officially recognized Indigenous peoples accounting for less than 0·2% of the total Russian population, with more than two-thirds living in rural locations.[5] There is limited research data regarding CVD among Russia's Indigenous populations.

North America

In North America, which has the most extensive data, Indigenous people have higher rates of cardiovascular risk factors, including smoking, diabetes, hypertension, dyslipidemia and obesity, which contribute to disparities in cardiovascular outcomes.[6–8] Additionally, Indigenous peoples in North America have been shown to have less access to cardiovascular diagnostics, percutaneous coronary intervention, and cardiac surgery, compared to non-Indigenous people[9–11], as well as less access to primary care providers and cardiovascular specialists, due in part to the rural and remote regions of the country in which they have traditionally lived.[12]

Canadian data from the 1980s demonstrates rates of ischaemic heart disease were noted to be lower in First Nations individuals than non-Indigenous individuals.[13,14] However, a random population-based study from 2001 demonstrated that First Nations individuals living on reserve had a rate of CVD that was almost 2·5 times higher than the non-Indigenous population.[7] Subsequently in the 2000s-2010s, rates of CVD declined in the First Nations population, but at a slower rate than the general population, resulting in a widening gap of CVD prevalence and mortality.[14,15] CVD affects American Indian individuals at younger ages than non-Indigenous Americans, with over one-third of CVD deaths occurring before the age of 65 years.[16,17] In addition to coronary heart disease, a higher prevalence of congestive heart failure, atrial fibrillation, stroke and peripheral arterial disease compared with the general US population has been reported.[6,18–21] Furthermore, because of colonization and intergenerational trauma, substance use rates (smoking and alcohol in particular) are higher in some Indigenous populations[7,22,23], with smoking rates of American Indians approximately double that seen in Black and White Americans.[22]

In Mexico and Central America, acculturation and Westernized lifestyles have increased cardiovascular risk factors among Indigenous communities, such as the Yaqui in Mexico and Kuna in Panama.[24,25] Additionally, in Guatemala, where over 50% of the population is Indigenous Maya[26], there is substantial prevalence of modifiable CVD risk factors in the Indigenous population, in particular hypertension, diabetes, obesity (among women), and smoking (among men).[27] Indigenous and Afro-descendant communities, comprising 11% (over 800 distinct groups) and 20% of the total population in Central America, respectively, face substantial disparities in health status and access to healthcare services, compared to the general population.[28]

References

1. International Work Group for Indigenous Affairs. The Indigenous World 2024: Sápmi [Internet]. IWGIA. 2024 [cited 2024 Oct 28]. Available from: https://www.iwgia.org/en/sapmi/5392-iw-2024-s%C3%A1pmi.html

2. Siri SRA, Eliassen BM, Broderstad AR, Melhus M, Michalsen VL, Jacobsen BK, et al. Coronary heart disease and stroke in the Sami and non-Sami populations in rural Northern and Mid Norway-the SAMINOR Study. Open Heart. 2020 May;7(1):e001213.

3. Fodor JG, Helis E, Yazdekhasti N, Vohnout B. "Fishing" for the Origins of the "Eskimos and Heart Disease" Story: Facts or Wishful Thinking? Canadian Journal of Cardiology. 2014 Aug;30(8):864–8.

4. Bjerregaard P, Kue Young T, Hegele RA. Low incidence of cardiovascular disease among the Inuit—what is the evidence? Atherosclerosis. 2003 Feb;166(2):351–7.

5. International Work Group for Indigenous Affairs. The Indigenous World 2024: Russia [Internet]. IWGIA. 2024 [cited 2024 Oct 28]. Available from: https://www.iwgia.org/en/russia/5394-iw-2024-russia.html

6. Eberly LA, Shultz K, Merino M, Brueckner MY, Benally E, Tennison A, et al. Cardiovascular Disease Burden and Outcomes Among American Indian and Alaska Native Medicare Beneficiaries. JAMA Netw Open. 2023 Sep 5;6(9):e2334923–e2334923.

7. Anand SS, Yusuf S, Jacobs R, Davis AD, Yi Q, Gerstein H, et al. Risk factors, atherosclerosis, and cardiovascular disease among Aboriginal people in Canada: the Study of Health Assessment and Risk Evaluation in Aboriginal Peoples (SHARE-AP). Lancet. 2001/10/13 ed. 2001 Oct 6;358(9288):1147–53.

8. Schultz A, Dahl L, McGibbon E, Brownlie J, Cook C, Elbarouni B, et al. Differences in coronary artery disease complexity and associations with mortality and hospital admissions among First Nations and non-First Nations patients undergoing angiography: a comparative retrospective matched cohort study. CMAJ Open. 2020 Nov 2;8(4):E685–94.

9. Kruse G, Lopez-Carmen VA, Jensen A, Hardie L, Sequist TD. The Indian Health Service and American Indian/Alaska Native Health Outcomes. Annual Review of Public Health. 2022 Apr 5;43(1):559–76.

10. Wei-Randall HK, Davidson MJ, Jin J, Mathur S, Oliver L. Acute myocardial infarction hospitalization and treatment: Areas with a high percentage of First Nations identity residents. Health Rep. 2013 Jul;24(7):3–10.

11. Li R, Choi BG. Native Americans have comparable transcatheter aortic valve replacement outcomes but higher stroke and venous thromboembolism after surgical aortic valve replacement. Cardiovascular Revascularization Medicine. 2024 May;62:11–7.

12. Schultz A, Dahl L, McGibbon E, Brownlie J, Cook C, Elbarouni B, et al. Health Outcome and Follow-up Care Differences Between First Nation and Non-First Nation Coronary

Angiogram Patients: A Retrospective Cohort Study. Canadian Journal of Cardiology. 2018 Oct;34(10):1333–40.

13. Shah BR, Hux JE, Zinman B. Increasing rates of ischemic heart disease in the native population of Ontario, Canada. Arch Intern Med. 2000/06/29 ed. 2000 Jun 26;160(12):1862–6.

14. Young TK. Cardiovascular Health among Canada's Aboriginal Populations: A Review. Heart, Lung and Circulation. 2012 Oct;21(10):618–22.

15. Chu A, Han L, Roifman I, Lee DS, Green ME, Jacklin K, et al. Trends in cardiovascular care and event rates among First Nations and other people with diabetes in Ontario, Canada, 1996-2015. CMAJ. 2019 Nov 25;191(47):E1291–8.

16. Stouffer JA, Hendrickson MJ, Arora S, Vavalle JP. Contemporary Trends in Acute Myocardial Infarction in the American Indian/Alaska Native U.S. Population, 2000 to 2018. The American Journal of Cardiology. 2023 May;194:34–9.

17. Gonuguntla K, Sattar Y, Iqbal K, Sharma A, Yadav R, Alharbi A, et al. Trends in Premature Mortality from Acute Myocardial Infarction in American Indians/Alaska Natives in the United States from 1999 to 2020. American Journal of Cardiology. 2024 Feb;213:72–5.

18. Ariss RW, Minhas AMK, Lang J, Ramanathan PK, Khan SU, Kassi M, et al. Demographic and Regional Trends in Stroke-Related Mortality in Young Adults in the United States, 1999 to 2019. J Am Heart Assoc. 2022/09/08 ed. 2022 Sep 20;11(18):e025903–e025903.

19. Baxter AR, Jacobowitz GR, Guo Y, Maldonado T, Adelman MA, Berger JS, et al. Increased Prevalence of Moderate and Severe Peripheral Arterial Disease in the American Indian (AI)/Alaskan Native (AN) Population; a Study of 96,000 AI/AN. Annals of Vascular Surgery. 2017 Jan;38:177–83.

20. Balabanski AH, Dos Santos A, Woods JA, Mutimer CA, Thrift AG, Kleinig TJ, et al. Incidence of Stroke in Indigenous Populations of Countries With a Very High Human Development Index. Neurology. 2024 Mar 12;102(5).

21. Heath T, Shrishail N, Wong KH, Johnston KC, Sharma R, Ney JP, et al. Trends in American Indian/Alaskan native self-reported stroke prevalence and associated modifiable risk factors in the United States from 2011-2021. J Stroke Cerebrovasc Dis. 2024/03/08 ed. 2024 Jun;33(6):107650–107650.

22. Breathett K, Sims M, Gross M, Jackson EA, Jones EJ, Navas-Acien A, et al. Cardiovascular Health in American Indians and Alaska Natives: A Scientific Statement From the American Heart Association. Circulation. 2020/05/28 ed. 2020 Jun 23;141(25):e948–59.

23. Sikorski C, Leatherdale S, Cooke M. Tobacco, alcohol and marijuana use among Indigenous youth attending off-reserve schools in Canada: cross-sectional results from the Canadian Student Tobacco, Alcohol and Drugs Survey. Health Promot Chronic Dis Prev Can. 2019 Jun;39(6–7):207–15.

24. Rodríguez-Morán M, Guerrero-Romero F, Brito-Zurita O, Rascón-Pacheco RA, Pérez-Fuentes R, Sánchez-Guillén MC, et al. Cardiovascular Risk Factors and Acculturation in Yaquis and Tepehuanos Indians from Mexico. Archives of Medical Research. 2008 Apr;39(3):352–7.

25. Hanna DR, Walker RJ, Smalls BL, Campbell JA, Dawson AZ, Egede LE. Prevalence and correlates of diagnosed and undiagnosed hypertension in the indigenous Kuna population of Panamá. BMC Public Health. 2019 Jun 28;19(1):843–843.

26. Mamo D. The Indigenous World 2022 [Internet]. IWGIA. 2022 [cited 2025 Jan 28]. Available from: https://iwgia.org/en/resources/publications/5503-the-indigenous-world-2022.html

27. Steinbrook E, Flood D, Barnoya J, Montano CM, Miller AC, Rohloff P. Prevalence of Hypertension, Diabetes, and Other Cardiovascular Disease Risk Factors in Two Indigenous Municipalities in Rural Guatemala: A Population-Representative Survey. Glob Heart. 2022;17(1):82.

28. Antonio G. Racial and ethnic disparities in Latin America and the Caribbean: a literature review. Diversity in Health and Care. 2010;7(2):115–28.
